# Response Predictivity to Neoadjuvant Therapies in Breast Cancer: A Qualitative Analysis of Background Parenchymal Enhancement in DCE-MRI

**DOI:** 10.3390/jpm11040256

**Published:** 2021-04-01

**Authors:** Daniele La Forgia, Angela Vestito, Maurilia Lasciarrea, Maria Colomba Comes, Sergio Diotaiuti, Francesco Giotta, Agnese Latorre, Vito Lorusso, Raffaella Massafra, Gennaro Palmiotti, Lucia Rinaldi, Rahel Signorile, Gianluca Gatta, Annarita Fanizzi

**Affiliations:** 1 Struttura Semplice Dipartimentale di Radiodiagnostica Senologica, I.R.C.C.S. Istituto Tumori “Giovanni Paolo II”, Viale Orazio Flacco 65, 70124 Bari, Italy; d.laforgia@oncologico.bari.it; 2 Unità Operativa Complessa di Radiologia–Senologia–P.O. San Paolo–ASL Bari, Via Caposcardicchio, 70123 Bari, Italy; angela.vestito@asl.bari.it (A.V.); m.lasciarrea@gmail.com (M.L.); 3 Struttura Semplice Dipartimentale di Fisica Sanitaria, I.R.C.C.S. Istituto Tumori “Giovanni Paolo II”, Viale Orazio Flacco 65, 70124 Bari, Italy; mariac.comes@libero.it (M.C.C.); a.fanizzi@oncologico.bari.it (A.F.); 4 Struttura Semplice Dipartimentale di Chirurgia Senologica, I.R.C.C.S. Istituto Tumori “Giovanni Paolo II”, Viale Orazio Flacco 65, 70124 Bari, Italy; sergiodiotaiuti@gmail.com; 5 Unità Operativa Complessa di Oncologia Medica, I.R.C.C.S. Istituto Tumori “Giovanni Paolo II”, Viale Orazio Flacco 65, 70124 Bari, Italy; francescogiotta@libero.it (F.G.); a.latorre@oncologico.bari.it (A.L.); vitolorusso@me.com (V.L.); 6 Struttura Semplice Dipartimentale di Oncologia Medica per la Presa in Carico Globale del Paziente Oncologico “Don Tonino Bello”, I.R.C.C.S. Istituto Tumori “Giovanni Paolo II”, Viale Orazio Flacco 65, 70124 Bari, Italy; gennaropalmiotti@hotmail.it (G.P.); l.rinaldi@oncologico.bari.it (L.R.); 7 Dipartimento di Chimica, Università degli Studi di Bari “Aldo Moro”, Via E. Orabona 4, 70125 Bari, Italy; rahel.signorile@gmail.com; 8 Department of Precision Medicine, University of Campania “Luigi Vanvitelli”, 81100 Naples, Italy; ggatta@sirm.com

**Keywords:** background parenchymal enhancement, breast MRI, breast cancer, neoadjuvant chemotherapy, fibro glandular tissue

## Abstract

Background: For assessing the predictability of oncology neoadjuvant therapy results, the background parenchymal enhancement (BPE) parameter in breast magnetic resonance imaging (MRI) has acquired increased interest. This work aims to qualitatively evaluate the BPE parameter as a potential predictive marker for neoadjuvant therapy. Method: Three radiologists examined, in triple-blind modality, the MRIs of 80 patients performed before the start of chemotherapy, after three months from the start of treatment, and after surgery. They identified the portion of fibroglandular tissue (FGT) and BPE of the contralateral breast to the tumor in the basal control pre-treatment (baseline). Results: We observed a reduction of BPE classes in serial MRI checks performed during neoadjuvant therapy, as compared to baseline pre-treatment conditions, in 61.3% of patients in the intermediate step, and in 86.7% of patients in the final step. BPE reduction was significantly associated with sequential anthracyclines/taxane administration in the first cycle of neoadjuvant therapy compared to anti-HER2 containing therapies. The therapy response was also significantly related to tumor size. There were no associations with menopausal status, fibroglandular tissue (FGT) amount, age, BPE baseline, BPE in intermediate, and in the final MRI step. Conclusions: The measured variability of this parameter during therapy could predict therapy effectiveness in early stages, improving decision-making in the perspective of personalized medicine. Our preliminary results suggest that BPE may represent a predictive factor in response to neoadjuvant therapy in breast cancer, warranting future investigations in conjunction with radiomics.

## 1. Introduction

In breast imaging, there are many diagnostic techniques that, with various modalities and different performance levels, detect breast cancer early and estimate residual disease. The best known are mammography (MG), ultrasound (US), and breast magnetic resonance imaging (MRI). New imaging techniques such as digital breast tomosynthesis (DBT), contrast-enhancement spectral mammography (CESM), and automated breast ultrasound (ABUS) have recently been introduced.

Establishing a correct response to oncological therapies is particularly complicated in exclusively morphological techniques such as mammography, especially in findings such as distortions, microcalcifications, or spiculated masses in which the quotas attributable to fibrosis, desmoplastic reaction, and neoplastic vital residue often appear indistinguishable [1]. This problem is overcome with contrast techniques, MRI and CESM, that emphasize the tumor areas with active neoangiogenesis by separating them from the areas with fibrosis and necrosis, which better highlights the complete pathological response (pCR) [1,2]. MRI is an older, more widespread and studied technique than CESM. It is multiparametric, three-dimensional, and has no exposure to X-rays, which is why it is of preferential use compared to the latter, even if the performances between the latter two techniques are comparable [2]. Some authors suggest that MRI is more accurate than other imaging modalities for assessing residual disease after neoadjuvant chemotherapy (NAC) [1].

Studying tumor microenvironments represents the next frontier in oncological therapies, as it will help quantify the evolution of diseases in the coming years and possibly predict tumor recurrence or progression [3].

In this context, an emerging parameter in breast imaging, which could represent the microenvironment characteristics, is the background of parenchymal enhancement (BPE). BPE is defined as the normal background impregnation of the gland after gadolinium injection [4,5,6] related to the physiological vascularization and perfusion of breast tissue [7,8,9,10,11].

Several studies detected a high value of BPE as a decisive predictive factor of breast cancer risk, regardless of other typical factors such as breast density. Moreover, BPE is frequently associated with a higher incidence of ductal carcinoma in situ [12,13,14,15]. A more evident BPE around the tumor, associated with a high T stage, represents a potential independent factor related to shorter disease-free survival [16].

A moderate to high BPE may also impact the diagnostic sensitivity and accuracy of an MRI test [17,18,19] Therefore, BPE has been recently included in the MRI’s Lexicon ACR-BIRADS [20,21], which suggests reporting distribution and intensity through a subdivision into four classes: minimal (BPE <25% of glandular tissue demonstrating enhancement), mid (25–50% enhancement), moderate (50–75% enhancement)), and marked (>75% enhancement).

According to some previous studies, BPE is considered to be a potential predictor of response to neoadjuvant therapy [22,23,24,25,26]. In particular, the reduction in BPE intensity over time, measured by MRI in the disease-free breast, depends on the menopausal status and appears to be more associated with pCR than NAC. However, it could also be related to ovarian suppression caused by chemotherapy and to the better vascularization and vasal permeability of certain types of breasts. This type of assessment is still being studied; particularly, the information concerning pre-treatment BPE intensity [4,6,22,26,27,28,29,30] appears to be controversial. For the reasons mentioned above, BPE is considered an interesting diagnostic and prognostic indicator in breast oncology, yet some aspects are still not fully defined and are deserving of further studies.

This evaluation can be carried out by qualitative method, i.e., by the visual evaluation of one or more radiologists, or by automatic or semi-automatic quantitative methods [25,30,31]. It is important to note that assessing BPE using an objective and automated evaluation method may achieve opposite results [30,31]. Therefore, more in-depth studies are necessary along with more standardized methods to validate them.

Another aspect of great interest is the effectiveness of specific neoadjuvant treatments that depend on the cancer molecular subtype and treatment [31]. Since quantitative methods are being developed and are not yet standardized, in this preliminary study, we examine the qualitative assessment of BPE, which is more standardized and suitable in current clinical practice.

This work aims to analyze BPE as a potential prognostic, predictive factor when evaluating the effect of oncological neoadjuvant therapies on the basis of qualitative criteria.

## 2. Materials and Methods

### 2.1. Experimental Data

From 1 September 2015 to 31 July 2020, we collected data and MRI images of 80 consecutive patients suffering from breast cancer. These patients were subjected to at least two diagnostic steps in the course of neoadjuvant oncological therapy and treated at the Istituto Tumori “Giovanni Paolo II” of Bari.

Patients were recruited according to the following inclusion criteria:

- Patients over the age of 18 with a histological diagnosis of infiltrative breast cancer of various histology (ductal, lobular, other) and various molecular subtypes, with clinical-stage II-III sec. The Classification of Malignant Tumours TNM [1] 8^ edition received a clinical indication for neoadjuvant chemotherapy.
- Carrying out at least two MRI evaluations, i.e., before the onset of oncology treatment and after three months of therapy.
- Written informed consent to procedures and use of data.

The exclusion criteria were as follows:

- Absence of at least two MRI controls in therapy.
- Failure to consent to the procedures and use of data.

This study was approved by the Scientific Board of the Istituto Tumori “Giovanni Paolo II” and carried out in the manner prescribed by the Helsinki Statement. On the basis of our regulation on retrospective studies, all patients who gave consent to use the data for scientific purposes were recruited.

### 2.2. Molecular Subtype Characterization and Neoadjuvant Cancer Therapy

In this study, we retrospectively analyzed data from a sample of 80 patients diagnosed with breast cancer from stage I to stage III, according to the TNM 8^ edition. Candidates received neoadjuvant chemotherapy. The different molecular histotypes were also defined by an immunohistochemical assessment of the expression of estrogen and progesterone receptors, as well as ki67 and Her-2 status, according to the molecular classification of the St. Gallen Consensus Conference of 2013 [32]. They were, therefore, divided into five main groups: luminal A-like; luminal B-like HER2-negative; luminal B-like HER2-positive; HER2-positive non-luminal; triple-negative. The status of HER2 was defined according to the international ASCO-CAP [3] the American Society of Clinical Oncology and the College of American Pathologists guidelines [33,34].

NAC administration followed a specific scheme. For patients with breast cancer, it went as follows: luminal A-like, luminal B-like, HER2-negative, and triple-negative. Next, sequential chemotherapy with four cycles of anthracycline (adriamycin or epirubicin) was combined with cyclophosphamide at three-week intervals for three months, followed by a further three months of therapy with taxanes (i.e., docetaxel for four cycles at three-week intervals or weekly paclitaxel for 12 consecutive weeks). For HER2 positive patients, a combination of taxanes and trastuzumab was used.

The patients were subjected to MRI timed controls. The first one, defined as the “basaline”, occurred before the beginning of the NAC. The second, defined as “step I or the middle step”, was performed after at least three months of treatment, i.e., after the first four rounds of therapy. The third MRI control, defined as “step II or the final step”, was performed after the end of sequential chemotherapy with taxanes (whether or not combined with anti-HER2 therapy according to the molecular subgroup).

In accordance whit RECIST Criteria [35], pathological responses to therapy were assessed by considering four possible outcomes:

- Complete Response (CR): Disappearance of all target lesions. Any pathological lymph nodes (whether target or non-target) must have reduction in short axis to <10 mm;
- Partial Response (PR): At least a 30% decrease in the sum of diameters of target lesions, taking as reference the baseline sum diameters;
- Progressive Disease (PD): At least a 20% increase in the sum of diameters of target lesions, taking as reference the smallest sum on study (this includes the baseline sum if that is the smallest on study). In addition to the relative increase of 20%, the sum must also demonstrate an absolute increase of at least 5 mm. (note: the appearance of one or more new lesions is also considered progression);
- Stable Disease (SD): Neither sufficient shrinkage to qualify for PR nor sufficient increase to qualify for PD, taking as reference the smallest sum diameters while on study.

The pathological response category was attributed after surgery and evaluated respect to the initial size of the tumor before treatment observed in MRI by considering the RECIST criteria.

### 2.3. Protocol for Patient Data Collection in Neoadjuvant Therapy

Three radiologists dedicated to breast imaging with 10 or more years of experience with breast MRI examined, in triple-blind modality, the available MRI. They identified the portion of fibroglandular tissue (FGT) and BPE of the contralateral breast to the tumor in the basal control pre-treatment (baseline). In the following steps, they performed these after three months of therapy (intermediate step) and at the end of treatment before surgery (final step). All 80 patients presented at least the basal and intermediate steps to the final observation. Each radiologist independently acquired the BPE and the prevailing class was considered valid. The divergent cases in the evaluations came among operators from a class discussed during a dedicated multidisciplinary meeting to gain consensus.

Investigations were carried out using MRI 1.5 Tesla equipment (Achieva, Philips Medical Systems, Best, The Netherlands). Patients lay prone on dedicated coils while radiologists examined their breasts. For premenopausal patients or women who had still not undergone ovarian suppression, the study was performed in the second week of the cycle. The sequences used were as follows: T1 inversion recovery (STIR), diffusion (DWI), turbo-spin-echo (TSE) without contrast administration, and T1 3D-DCE (6 dynamic acquisitions of 1.5 mm^3^ a voxel isotropic of 60 s each, one before and five after intravenous administration, with automatic paramagnetic contrast agent injector at a dose of 0.1 mmol/kg body weight and a flow of 2–2.5 mL/s, followed by 20 mL of saline solution). At the end of the acquisition of dynamic T1 sequences, an automatic subtraction process between post-contrast and pre-contractual images (of the same sequence) allowed for the detection of images subtracted with an emphasis on areas of pathological enhancement in the breast. The investigation was then completed with the creation of I/T [5] enhancement curves and a maximum intensity projection vascular map (MIP).

**Figure 1:**
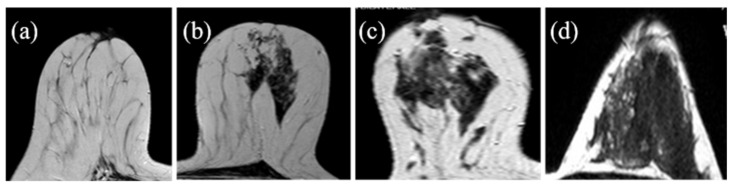
Fibroglandular tissue (FGT): (**a**)almost entirely fat; (**b**) scattered fibroglandular tissue; (**c**) heterogeneous fibroglandular tissue; (**d**) extreme fibroglandular tissue.

**Figure 2:**
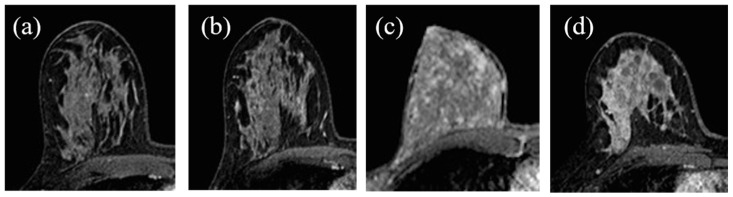
Background parenchymal enhancement (BPE): (**a**) minimal; (**b**) mild; (**c**) moderate; (**d**) marked.

The operators’ assessments of FGT were carried out on the T2 morphological sequences and the first post-contrast acquisition of the dynamic sequence; otherwise, BPE evaluations were carried out on the first post-gadolinium dynamic sequence, as based on the literature [23,34]. FGT and BPE were classified according to ACR BIRADS [7] criteria in four groups: FGT in almost entirely fat (a), scattered fibro glandular tissue (b), heterogeneous fibro glandular tissue (c), extreme fibro glandular tissue (d), and BPE in minimal (I), mild (II), moderate (III), marked (IV), symmetric, or asymmetric. Examples of this classification are shown in Figure 1 and Figure 2.

**Figure 3:**
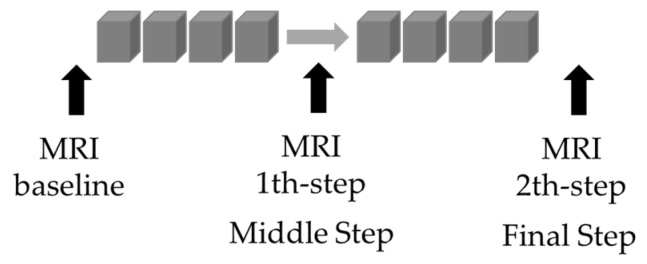
Flow chart breast magnetic resonance imaging (MRI) exam procedure and neoadjuvant therapy.

For the study, we selected and differentiated patients who underwent therapy with anthracyclines and cyclophosphamide in the first three months of treatment and only taxanes (with and without anti-HER2+therapy) in the following three months until the end (Figure 3).

### 2.4. Statistical Analysis

The Chi-square test was used to evaluate the significant association between two categorical variables, such as BPE, FGT, the type of therapy, molecular subtype, the menopausal state, response to therapy, and BPE reduction.

Patients were classified into five groups of cancer subtypes according to the St. Gallen International Expert Consensus on the Primary Therapy of Early Breast Cancer 2013 [32]: luminal A, luminal B HER2 negative, luminal B HER2 positive, HER2 positive non-luminal, and triple-negative.

BPE reduction was assessed considering, for each patient, the transition to a lower class after three months of therapy (BPE middle step) or the end (BPE final step) compared to the basal assessment (BPE baseline).

Cohen’s kappa statistics were used to assess the inter-observer agreement for classifying BPE and FGT.

A result was considered significant when the *p*-value was less than 0.05. All calculations were performed using SPSS statistical software.

## 3. Results

Table 1 summarizes the characteristics of the analyzed samples. In total, 80 patients with histologically proven breast cancer aged between 31 and 80 years (with average, first, and fourth quartiles of 49.0, 43.3, and 62.8 years, respectively). All patients had at least two MRI evaluations during neoadjuvant therapy, but only 37.5% (30/80) of patients performed all three steps.

The operators’ evaluation of the FGT and BPE in the three resonance steps had a good level of agreement with a Cohen’s kappa value of about 0.55 for each comparison that was significantly different from 0 (*p*-values Cohen’s kappa test of each comparison <0.05). However, as described above, the discordant cases over a class (*n* = 9) were then reassessed and a general consensus was achieved. In the remaining cases, the most represented class was acquired.

Menopausal status was significantly associated with FGT (Table 2) and baseline BPE (Table 3). Indeed, patients with low FGT (I, II) and low baseline BPE (minimal, mild) tended to be post-menopausal patients, while patients with high FGT (IV) and baseline BPE (moderate, marked) were pre-menopausal. This was probably related also to the age factor with which the FGT and baseline BPE were significantly associated (*p*-value Kruskal–Wallis test < 0.05).

As shown in Table 4, after the middle-step MRI step, 79.4% of patients with a moderate baseline BPE showed minimal BPE, while 54.4% of patients with a mild baseline BPE showed moderate BPE. Although only 37.5% of the patients in the sample performed the final step MRI, 93.3% of patients with a moderate baseline BPE exhibited a significant class reduction towards the minimal BPE.

Specifically, after the first three months of NAC, 49 patients (61.3%) showed a reduction in the BPE class, while 31 (38.7%) patients maintained the starting class. Among the 30 patients who also performed the final MRI step, 26 (86.7%) showed a class reduction compared to the baseline BPE. BPE reduction was associated with the baseline BPE class; in particular, a reduction was observed when BPE was moderate/mild (Table 5).

BPE reduction was significantly associated with the chemotherapy administrated in the first round of neoadjuvant therapy (Table 6). A significant BPE reduction was observed in patients treated with anthracyclines, taxanes, or both. On the other hand, there was no significant association concerning other characteristics examined.

The response to therapy was associated with the size of the lesions. The initial size of the lesion for patients who had a complete response was on average 17.5 mm, while for those who had a partial response was 38.0 mm (Table 7). The response to therapy was not associated with the menopausal status, age, and molecular subtype of the tumor.

**Figure 4:**
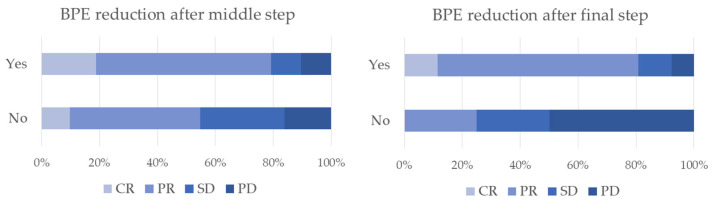
Distribution of BPE reduction after middle and final step respect to neoadjuvant chemotherapy (NAC).

The response to NAC was not associated with FGT, BPE baseline, as well as BPE in the middle and final step (Table 8). We counted the cases that found BPE reduction to be at least one BIRADS class at the middle and/or final step MRI compared to the baseline. The response to therapy was found to be significantly associated with BPE reduction (Figure 4). Indeed, 61.2% and 18.4% of patients who showed a reduction in BPE after the first three months of NAC had a partial and a complete response; only 45.2% of patients who did not show a reduction in BPE after the first three months of NAC had a partial response, while 29.0% with the disease remained stable (Table 8). Therefore, sensitivity and specificity for BPE reduction predicted a partial or complete response was 77.6% and 45.2%, respectively, with positive and negative predictive values of 55 and 24, respectively. In addition, there was no significant association between a response to therapy or dosing regimen (Table 9).

**Figure 5:**
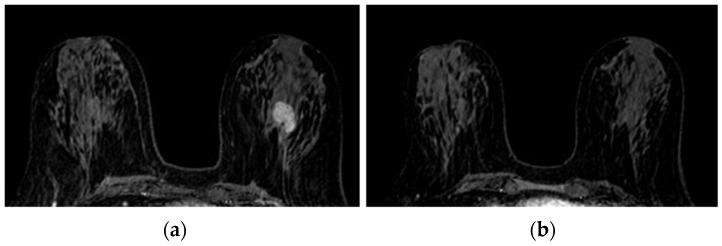
Complete pathological response (pCR): 2,5 cm deep retroareolar tumor on the left breast in the pre-treatment baseline survey ((**a**) triple negative) no longer visible at the end of the therapy cycles (**b**). Reduction of a BPE class (moderate to mild) is observed.

**Figure 6:**
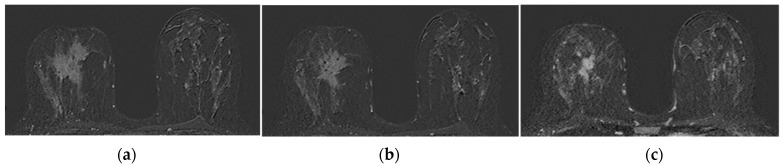
Partial pathological response (PR): large spiculated neoplastic lesion (4.5 cm) in right retroareolar (HER2 +) with moderate contralateral BPE in the pretreatment baseline image (**a**). Progressive reduction of lesion diameter is observed to 50% in step I MRI (**b**) and to 75% in step II MRI (**c**) without significant changes in BPE.

**Figure 7:**
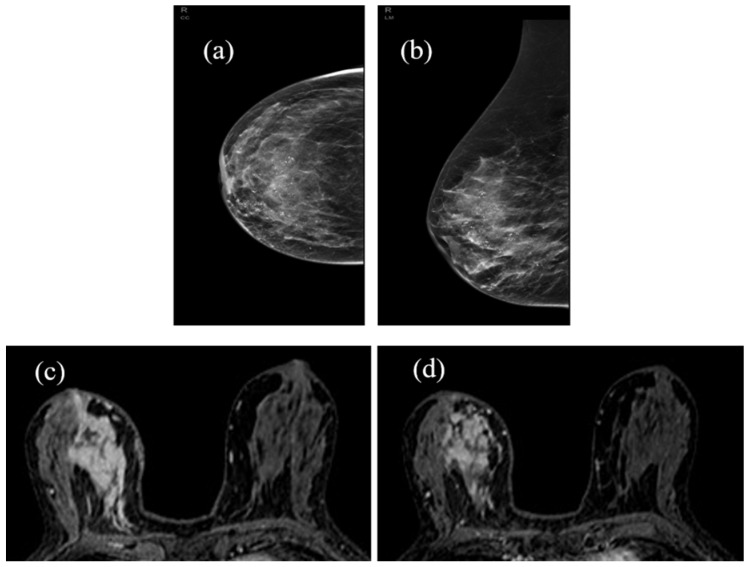
Stable disease (SD): Mammography shows the presence of a large area of granular microcalcifications in the right lower inner quadrant, unchanged during NAC (**a**,**b**). In MRI, it is evident in the same large area of pathological enhancement (**c**) that does not show significant changes in the extent and activity at the end of therapy. The BPE also appears unchanged (**d**). Histological type: invasive and in situ non-special type carcinoma (ductal).

Figure 5, Figure 6 and Figure 7 show three different examples of responses to cancer therapies according to the RECIST criteria.

## 4. Discussion

This study evaluated the possible correlations between changes in BPE (pre-treatment, after the first three months, and at the end of the treatment), FGT, menopausal state, therapies administered, molecular type, and the size of the tumor in breast cancer patients in neoadjuvant oncological treatment.

Menopausal status was associated significantly with FGT and baseline BPE, meaning that patients with both FGT and baseline BPE tended to be post-menopausal, whereas patients with high FGT and a higher baseline BPE were more likely pre-menopausal. These findings are naturally related to the age factor with which FGT and baseline BPE are significantly associated.

FGT is the proportion of fibroglandular tissue present in MRI and resembles mammography density, a known independent risk factor of BP [13]. FGT is like density on mammography, it can regress with the passing of age and especially after menopause.

Furthermore, BPE, linked to the glandular tissue physiological permeation, depends on several factors including age, hormonal status, ongoing hormonal therapies, previous radiotherapy, and ovariectomy [4,6,7,8,9,10,11].

In a qualitative analysis, it is also essential to precisely establish the research criteria. Indeed, according to the ACR guidelines, analyses should be performed on the first dynamic sequence after gadolinium, even if some authors verified the reliability of the measurements, subtractions, or the first three post-gadolinium sequences in an automated form after consensus between two or more radiologists [8,17,23,24]. On the basis of these indications, the observational evaluation in this study was conducted in the first post-contrast dynamic.

The qualitative measurement of these class parameters was also subject to inter-intra-observer variability [25,30,31,36,37,38] and would require the consensus of three radiologists through BIRADS criteria [20,39]. Therefore, an independent qualitative analysis with three different operators was carried out in order to minimize variability. This allowed us to reduce the number of discordant cases beyond one class (9/80 patients), which was then collectively revaluated in order to acquire a consensus.

The results of this study show a statistically significant reduction of the BPE class during serial evaluation performed in the course of neoadjuvant therapy. The reduction in BPE was significantly correlated to the share of responding patients with a complete or partial response (CR and PR). This presents a reduction of at least one class of BPE after the middle step MRI was 79.6% and became substantially unchanged even in the post final step MRI (79.2%). Among non-responding patients or patients with stable/progressing disease (SD, PD), only 20.2% after the middle step and 17.7% after the final step showed a reduction of at least one class of BPE. BPE decreased during neoadjuvant therapy in 4/5 responding patients and in 1/5 non-responders. This is an important aspect in our study because, if confirmed in larger cohorts of patients with quantitative evaluations, BPE could be confirmed as a marker of predictive response to NAC, thus allowing for an early detection of responders in a therapy continuation and sending non-responders straight to surgery. This may result in the optimization of the therapeutic path, as well as significant economic savings for the healthcare system. The standardization of automatic detection methods and quantification of BPE through specific features could improve the results shortly, starting from the correlation with molecular subtypes, as it happens in CESM [40]; however, this correlation is also possible with qualitative observational methods [41]

BPE reduction is significantly related to the administration of taxanes or anthracyclines in the first round of NAC compared to anti-HER2+combined therapies, which is consistent with reports from other authors [31]. This aspect could be related to the combination of the anti-angiogenetic drug with common chemotherapeutic agents that, in addition to improving the tumor’s response during chemotherapy, could operate through the normalization of the tumor vascular function while it converts non-functional vessels into functional ones. This would allow for more chemotherapeutic agents to reach the tumor [30]. This would also explain the lower BPE reduction related to the preserved tissue perfusion and the consequent higher percentage of pCR (18.8%) in HER2+ tumors compared to tumors observed in the study.

The relatively low sample size in the various categories represents our study limitation, even though it is in line with other published works [26,42]

A second limit may be the retrospective nature of the study. From having analyzed only two MRI steps in a significant proportion of the sample, this limit could eventually be considered apparent since the most significant changes in BPE occurred in the first treatment phase, as reported in the literature [22,23,24,30]. Our study supported this consideration by highlighting the BPE class’s confirmation between the intermediate and final step, showing a significant concordance in the results.

Throughout the study, the concordance among the operators was high (*p*-values k kappa of Cohen test <0.05), which could result from common training and years of experience in breast imaging, specifically. This correlation could probably be reduced if operators carried out assessments with different training and years of seniority. This may be the subject of evaluation in future work.

## 5. Conclusions

The study evidenced BPE’s role in predicting the response of a tumor to NAC through an inter-observer qualitative analysis. Future studies based on automatic BPE quantification through specific features could lead to an additional minimization of the variability related to an inter-evaluation observer. If the results of this preliminary study are confirmed in studies with larger samples, then an early evaluation of BPE just after the first cycle could predict NAC response.

## Figures and Tables

**Table 1:** Characteristics of the 80 patients analyzed in the study.

Characteristics	Patients Number	
	Absolut Value	%
Age		
Median (1st-quartile; 3rd-quartile)	49.0 (43.3; 62.8)	
Baseline diameter of the lesion(mm)		
Median (1st-quartile; 3rd-quartile)	35.0 (23.5; 50.0)	
Menopausal status		
no(yes)	32 (48)	40.0 (60.0)
Molecular subtype		
Luminal A	8	10.0
Luminal B	17	21.3
HER2 positive	16	20.0
Triple Negative	21	26.4
First Cycle of therapy		
Anthraacycline	33	41.3
Taxanes	11	13.8
Anthracycline + taxanes	5	6.3
Taxanes+ tantiHER2 therapies	26	32.5
No Chemotherapy cycle	5	6.3
BPE baseline		
Minimal	19	23.2
Mild	22	26.8
Marked	5	6.1
NaN	-	-
BPE 1st-step (Middle step)		
Minimal	51	63.8
Mild	6	7.5
Moderate	22	27.5
Marked		
NaN	-	-
BPE 2st-step (Finale step)		
Minimal	21	25.6
Mild	1	1.2
Moderate	8	9.8
Marked	-	-
NaN	50	62.5
FGT		
I	6	7.5
II	41	51.3
III	16	20.0
IV	17	21.3
Response to NAC		
Complete Response (CR)	12	15.0
Partial Response (PR)	44	55.0
Stable Disease (SD)	14	17.5
Progressive Disease (PD)	10	12.5

**Table 2:** Absolute frequency distribution (percentage) of fibroglandular tissue (FGT) of patients undergoing neoadjuvant chemotherapy (NAC) compared to menopausal condition.

		Post-Menopausal Status		
		No	Yes	Total
FGT	I	0 (0.0%)	6 (100%)	6 (100%)
	II	11 (26.8%)	30 (73.2%)	41 (100%)
	III	8 (50.0%)	8 (50.0%)	16 (100%)
	IV	13 (76.5%)	4 (23.5%)	17 (100%)
	Total	32 (40.0%)	48 (60.0%)	80 (100%)

**Table 3:** Absolute frequency distribution (percentage) of baseline background parenchymal enhancement (BPE) of patients undergoing NAC compared to menopausal condition.

		Post-Menopausal Status		
		No	Yes	Total
BPE baseline	Minimal	3 (15.8%)	16 (84.2%)	19 (100%)
	Mild	15 (68.2%)	7 (31.8%)	22 (100%)
	Moderate	10 (29.4%)	24 (70.6%)	34 (100%)
	Marked	4 (80.0%)	1 (20.0%)	5 (100%)
	Total	32 (40.0%)	48 (60.0%)	80 (100%)

**Table 4:** Absolute frequency distribution (percentage) of BPE in the three resonance steps of patients undergoing NAC.

		BPE Middle Step (*)					
		Minimal	Mild	Moderate	Marked	NaN	Total
BPE baseline	Minimal	15 (78.9%)	1 (5.3%)	3 (15.8%)	-	-	19 (100%)
	Mild	6 (27.3%)	4 (18.2%)	12 (54.5%)	-	-	22 (100%)
	Moderate	27 (79.4%)	-	7 (20.6%)	-	-	34 (100%)
	Marked	3 (60.0%)	1 (20.0%)	-	1 (20.0%)	-	5 (100%)
	Total	51 (63.8%)	6 (7.5%)	22 (27.5%)	1 (1.3%)	-	80 (100%)
BPE baseline	Minimal	2 (66.7%)	-	1 (33.3%)	-	-	3 (100%)
	Mild	5 (50.0%)	-	5 (50.0%)	-		10 (100%)
	Moderate	14 (93.3%)	-	1 (6.7%)	-	-	15 (100%)
	Marked	-	1 (50.0%)	1 (50.0%)	-	-	2 (100%)
	Total	21 (70.0%)	1 (3.3%)	8 (6.7%)	-	2 (6.7%)	30 (100%)

* *p*-value Chi-square test < 0.05.

**Table 5:** Baseline BPE reduction distribution compared to BPE reduction after 1st-step and 2st-step NAC.

		BPE Reduction after Middle Step (*)			BPE Reduction after Final Step (*)		
		No	Yes	Total	No	Yes	Total
BPE baseline	Minimal	19 (61.3%)	-	19 (23.8%)	3 (75.0%)	-	3 (10.0%)
	Mild	4 (12.9%)	18 (36.7%)	22 (27.7%)	-	10 (138.5%)	10 (33.3%)
	Moderate	7 (22.6%)	27 (55.1%)	34 (42.3%)	1 (25.0%)	14 (53.8%)	15 (50.0%)
	Marked	1 (3.2%)	4 (8.2%)	5 (6.3%)	-	2 (7.7%)	2 (6.7%)
	Total	31 (100%)	49 (100%)	80 (100%)	4 (100%)	26 (100%)	30 (100%)

* *p*-value Chi-square test < 0.05.

**Table 6:** Distribution of BPE reduction compared to the type of the first cycle of NAC.

		BPE Reduction after Middle Step (*)		
		No	Yes	Total
First cycle therapy	Anthracycline	11 (33.3%)	22 (66.7%)	33 (100%)
	Taxanes	3 (27.3%)	8 (72.7%)	11 (100%)
	Anthracycline + taxanes	0 (0.0%)	4 (100%)	4 (100%)
	Taxanes+ antiHER2 therapies	16 (61.5%)	10 (38.5%)	26 (100%)
	No Chemotherapy cycle	1 (25%)	3 (75%)	4 (100%)
	Total	31 (39.2%)	48 (60.8%)	79 (100%)

* *p*-value Chi-square test < 0.05.

**Table 7:** Response distribution to NAC with respect to patients’ characteristics and molecular subtype.

	Characteristics	CR	PR	SD	PD	Total
	Age					
	Median	47.5	47.5	50.0	49.0	49.0
	(1st-quartile; 3rd-quartile)	(38.0; 61.0)	(43.0; 60.0)	(50.0; 69.0)	(48.3; 61.8)	(43.3; 62.8)
§	Baseline diameter of the lesion(mm)					
	Median	17.5	38.0	30.0	33.5	35.0
	(1st-quartile; 3rd-quartile)	(12.0; 27.0)	(28.0; 50.0)	(24.0; 43.0)	(28.4; 65.0)	(23.5; 50.0)
	Menopausal status					
	No	5 (15.6%)	19 (59.4%)	4 (12.5%)	4 (12.5%)	32 (100%)
	Yes	7 (14.6%)	25 (52.1%)	10 (20.8%)	6 (12.5%)	48 (100%)
	Molecular subtype					
	Luminal A	1 (12.5%)	4 (50.0%)	2 (25.0%)	1 (12.5%)	8 (100%)
	Luminal B	2 (11.8%)	14 (82.4%)	1 (5.9%)	0 (0.0%)	17 (100%)
	HER2 positive	3 (18.8%)	8 (50.0%)	3 (18.8%)	2 (12.5%)	16 (100%)
	Triple Negative	3 (13.6%)	12 (54.5%)	2 (9.1%)	5 (22.7%)	22 (100%)
	Triple Positive	3 (17.6%)	6 (35.3%)	6 (35.3%)	2 (11.8%)	17 (100%)

§ *p*-value *T* test < 0.05.

**Table 8:** Distribution of response to NAC with respect to BPE, FGT, and reduction of BPE after the middle and final step.

	Characteristics	CR	PR	SD	PD	Total
	BPE baseline					
	Minimal	2 (10.5%)	7 (36.8%)	6 (31.6%)	4 (21.1%)	19 (100%)
	Moderate	4 (11.8%)	22 (64.7%)	5 (14.7%)	3 (8.8%)	34 (100%)
	Mild	5 (22.7%)	11 (50.0%)	3 (13.6%)	3 (13.6%)	22 (100%)
	Marked	1 (20.0%)	4 (80.0%)	-	-	5 (100%)
	NaN	-	-	-	-	-
	BPE middle step					
	Minimal	9 (18.0%)	26 (52.0%)	9 (18.0%)	6 (12.0%)	50 (100%)
	Mild	1 (16.7%)	2 (33.3%)	1 (16.7%)	2 (33.3%)	6 (100%)
	Moderate	2 (9.1%)	14 (63.6%)	4 (18.2%)	2 (9.1%)	22 (100%)
	Marked	-	1 (100%)	-	-	1 (100%)
	NaN	-	-	-	-	-
	BPE final step					
	Minimal	2 (9.5%)	15 (71.4%)	1 (4.8%)	3 (14.3%)	21 (100%)
	Mild	-	1 (100%)	-	-	1 (100%)
	Moderate	1 (12.5%)	3 (37.5%)	3 (37.5%)	1 (12.5%)	8 (100%)
	Marked	-	-	-	-	-
	NaN	-	-	-	-	-
	FGT					
	I	-	4 (66.7%)	2 (33.3%)	-	6 (100%)
	II	4 (9.8%)	22 (53.7%)	9 (22.0%)	6 (14.6%)	41 (100%)
	III	3 (18.8%)	8 (50.0%)	1 (6.3%)	4 (25.0%)	16 (100%)
	IV	5 (29.4%)	10 (58.8%)	2 (11.8%)	-	17 (100%)
	NaN	-	-	-	-	-
*	BPE reduction after middle step					
	Yes	9 (18.4%)	30 (61.2%)	5 (10.2%)	5 (10.2%)	49 (100%)
	No	3 (9.7%)	14 (45.2%)	9 (29.0%)	5 (16.1%)	31 (100%)
*	BPE reduction after final step					
	Yes	3 (10.0%)	18 (69.2%)	3 (10.0%)	2 (7.7%)	26 (100%)
	No	-	1 (25.0%)	1 (25.0%)	2 (50.0%)	4 (100%)

* *p*-value Chi-square test < 0.05.

**Table 9:** Distribution of the response to the neoadjuvant therapy with respect to the dosing regimen of the neoadjuvant therapy performed.

First Cycle Therapy	CR	PR	SD	PD	Total
Anthraacycline	4 (12.1%)	24 (72.7%)	2 (6.1%)	3 (9.1%)	33 (100%)
Taxanes	1 (9.1%)	6 (54.4%)	2 (18.2%)	2 (18.2%)	11 (100%)
Anthracycline + taxanes	1 (20.0%)	4 (80.0%)	0	0	5 (100%)
Taxanes+ antiHER2 therapies	5 (19.2%)	7 (26.9%)	9(34.6%)	5 (19.2%)	26 (100%)
No Chemotherapy cycle	1 (20.0%)	3 (60.0%)	1 (20.0%)	0	5 (100%)

## Data Availability

The data presented in this study are available on request from the corresponding author. The data are not publicly available because are propriety of Istituto Tumori ‘Giovanni Paolo II’—Bari, Italy.
